# Aza- and Azo-Stilbenes: Bio-Isosteric Analogs of Resveratrol

**DOI:** 10.3390/molecules25030605

**Published:** 2020-01-30

**Authors:** Gérard Lizard, Norbert Latruffe, Dominique Vervandier-Fasseur

**Affiliations:** 1 Team Bio-PeroxIL, Biochemistry of the Peroxisome, Inflammation and Lipid Metabolism (EA7270), University of Bourgogne Franche-Comté, Inserm, 21000 Dijon, France; gerard.lizard@u-bourgogne.fr (G.L.); norbert.latruffe@u-bourgogne.fr (N.L.); 2 Team OCS, Institute of Molecular Chemistry of University of Burgundy (ICMUB UMR CNRS 6302), University of Bourgogne Franche-Comté, 21000 Dijon, France

**Keywords:** trans-resveratrol, aza-stilbene, azo-stilbene, bio-isosterism, structure-activity relationship

## Abstract

Several series of natural polyphenols are described for their biological and therapeutic potential. Natural stilbenoid polyphenols, such as trans-resveratrol, pterostilbene and piceatannol are well-known for their numerous biological activities. However, their moderate bio-availabilities, especially for trans-resveratrol, prompted numerous research groups to investigate innovative and relevant synthetic resveratrol derivatives. This review is focused on isosteric resveratrol analogs aza-stilbenes and azo-stilbenes in which the C=C bond between both aromatic rings was replaced with C=N or N=N bonds, respectively. In each series, synthetic ways will be displayed, and structural sights will be highlighted and compared with those of resveratrol. The biological activities of some of these molecules will be presented as well as their potential therapeutic applications. In some cases, structure-activity relationships will be discussed.

## 1. Introduction

**Figure 1:**
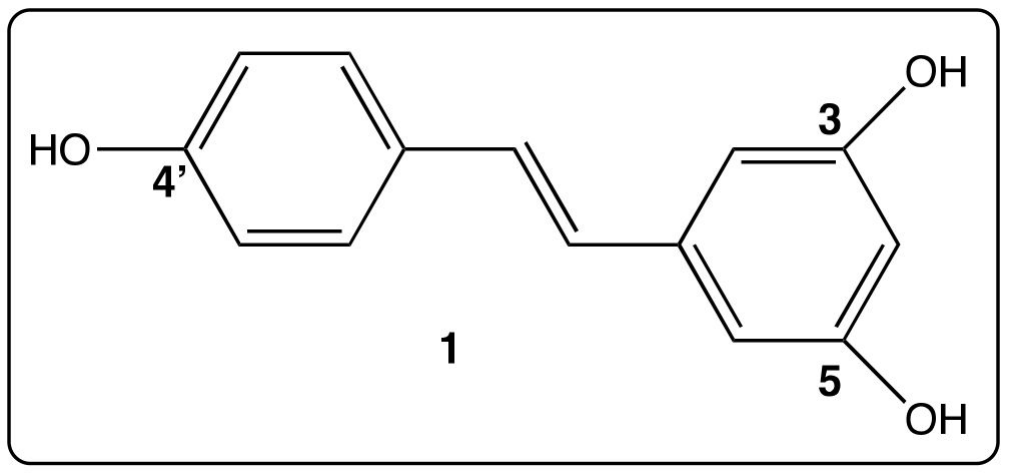
Structure of *trans*-Resveratrol (**1**).

Among natural polyphenolic compounds, polyphenolic stilbenoids hold an important place. They are widespread in a large number of plants. The leader of this series is a phytoalexin, trans-resveratrol (RSV) or 3,4′,5-trihydroxystilbene (1) (Figure 1) discovered in 1940 in Japan by M. Takaoka from *Veratrum grandiflorum* where its name comes from: VERATRum/resVERATRol [1].

This natural polyphenol is found in numerous species, for instance, in roots of Asiatic plant *Polygonum cuspidatum* [2], in several edible plants [3] especially in fruit including grapes where RSV is in the form of phytoalexin [4,5], and subsequently in red wine [6]. RSV is widely studied since the 90s. Because of its numerous biological activities, such as anti-oxidant [7], antitumoral [8], antiviral [9], and anti-inflammatory activities [10] and more recently due to its differentiating properties [11,12]. In addition, trans-resveratrol is a neuroprotective agent [13], and acts against platelet aggregation [14]. RSV is a sirtuin-activating compound (STAC) which may increase lifespan in metazoans (*Caenorhabditis elegans*, *Drosophila melanogaster*, mice) by a mechanism related with a caloric restriction [15,16,17]. Subsequently, this polyphenol is effective in treating metabolic disorders [18]. It is now recognized that a diet rich in these health-beneficial molecules allows a good level of health to be maintained [19].

However, despite its therapeutic potential, so far RSV could not be used as such in clinical trials because its quick metabolism and its weak bio-availability due to its low water solubility [20,21]. In order to enhance the bio-availability of RSV and subsequently, its therapeutic potency while keeping the hydroxylated stilbene scaffold, numerous research groups have synthesized an infinite number of RSV analogs and evaluated them for various biological activities. Several ways to modify RSV may be considered, for instance, transformation of phenolic functions in ester or ether functions [22,23,24], substitution with various groups on the phenyl rings [25,26,27,28] or by replacing a phenyl ring with an aromatic heterocycle [29,30] or with an organometallic cycle [27]. The concept of bio-isosterism may be applied in the case of RSV analogs [31]. Indeed, the C=C bond may be seen as a bridge between both phenyl rings, allowing an electronic delocalization on the whole molecule. This electronic feature plays a primordial role especially in anti-oxidant activity of the polyphenolic molecule. Thus, some RSV analogs have been designed by replacing the C=C bond with isosteric C=N or N=N bonds or with an aromatic ring [32,33,34,35]. Another way to overcome the problems related to the low water solubility of RSV is to load it into nanoparticles or liposomes [36,37,38,39]. Several reviews have focused on several series of RSV analogs especially natural RSV analogs [24], RSV analogs displaying pharmacological activities [40,41], multi-targeted drug RSV analogs [42] and RSV analogs with various substituents on both phenyl rings [28].

**Figure 2:**
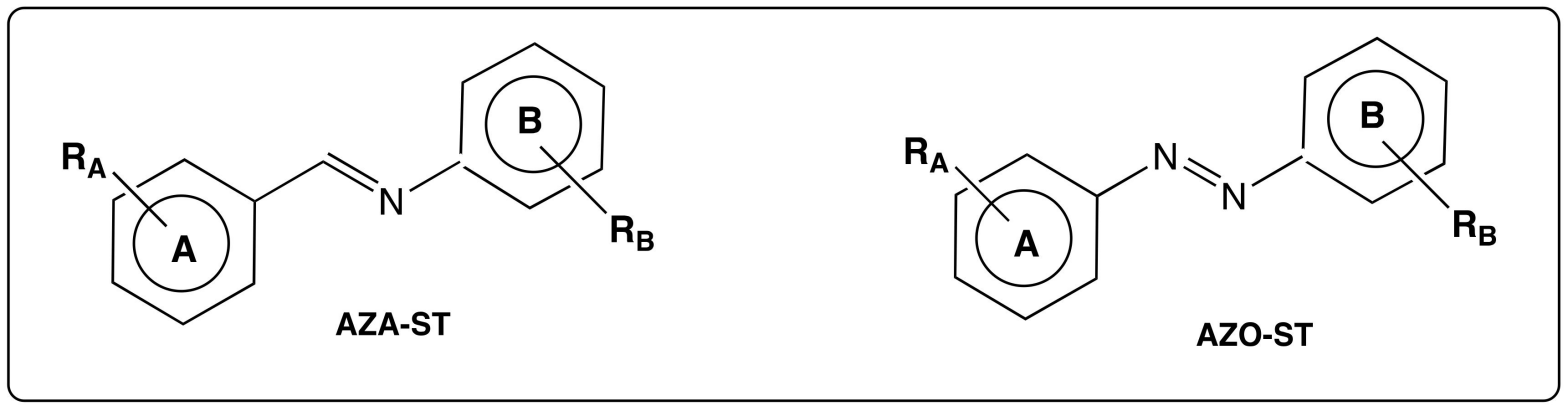
Structure of aza-stilbenes (AZA-ST) and azo-stilbenes (AZO-ST).

The aim of this review is to specifically focus on both aza-stilbenes (AZA-ST) and azo-stilbenes (AZO-ST) whose C=C bond is replaced by a C=N bond or N=N bond, respectively (Figure 2). Each series will be examined with respect to synthetic ways as well as structural sights and biological activities. In some cases, a relationship between the latter will be highlighted.

## 2. Structural and Synthetic Sights of AZA-ST and AZO-ST Compared with trans-RSV

### 2.1. Isosteric Features of C=N and N=N Bonds

Numerous RSV derivatives have been designed by keeping the original stilbenoid skeleton and changing the nature and/or the number of substituents on aromatic rings in order to enhance the bio-availability of the molecule, to better target the receptors, or to carry pharmacophore in the case of multi-targeted ligand derivatives [28]. These modifications usually require multi-steps syntheses which are often easy to carry out. The bio-isosterism concept is a discerning tool in drug design and may be used in the case of RSV analogs. According to Grimm’s hydride displacement law [31], nitrogen atom and CH group having the same number of valence electrons are isosters. Thus, the replacement of one or both CH in the double bond of stilbene by one or two nitrogen atoms is an easy synthetic way to obtain bio-isosters of RSV. In such isosters, the number of valence electrons does not change with respect to the parent molecule electronic environment of the stilbene scaffold and subsequently, the resulting RSV analogs may have biological activities similar to, or even superior to RSV.

### 2.2. Synthetic Pathways for Obtaining AZA-ST and AZO-ST

**Figure 3:**
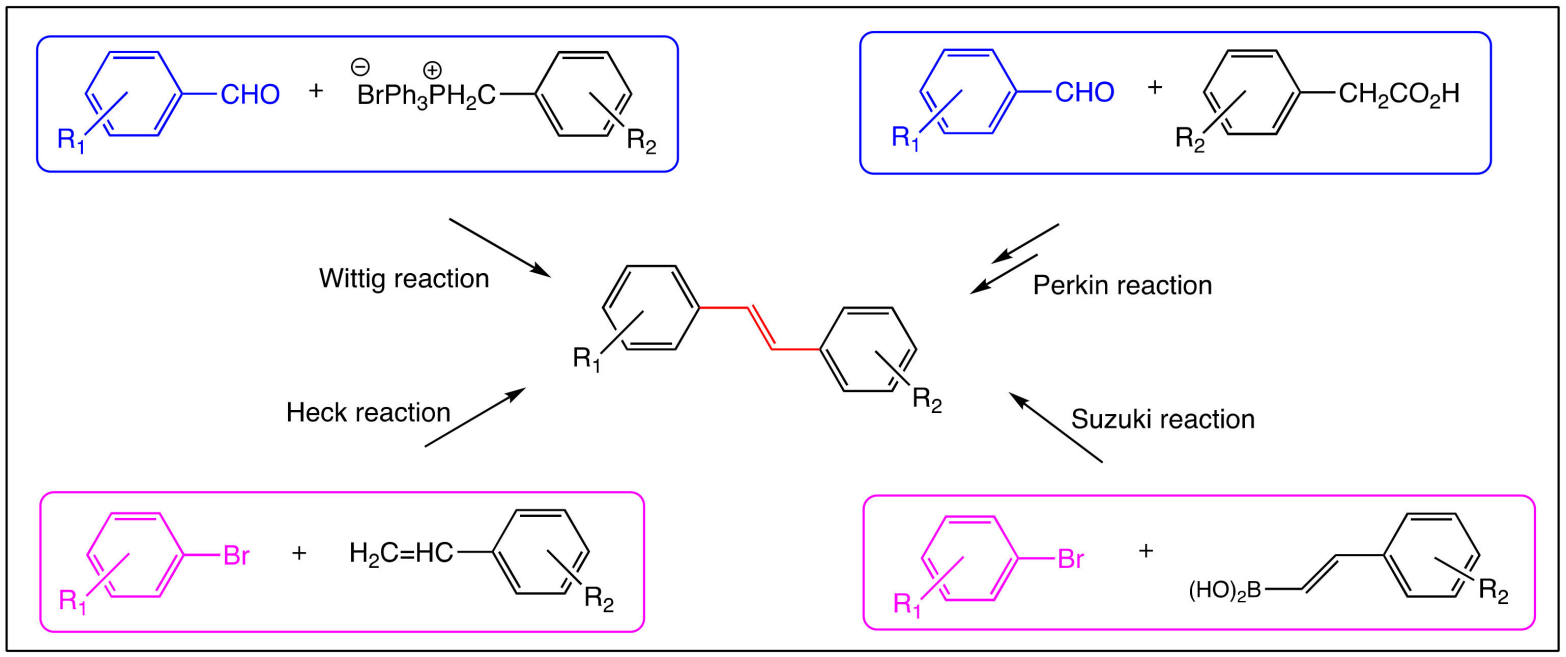
Principal synthetic methods for obtaining stilbene derivatives: Wittig method [25], Perkin method [43], Heck method [25] and Suzuki method [45].

Among the numerous synthetic ways to get RSV derivatives bearing a C=C bond, the key-step is always the formation of this linkage which, can be formed by different chemical methods including Perkin [43], Wittig [25], Horner-Wittig-Emmons [44], Heck [25] and Suzuki [45] reactions from starting aromatic aldehydes or aryl bromide (Figure 3). These methods are summarized in the figure below (Figure 3); some of them require protection steps of phenolic functions.

**Scheme 1:**
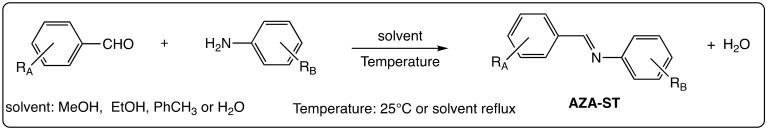
Synthetic methods for obtaining aza-stilbenes (AZA-ST).

Aza-stilbenes (AZA-ST) are commonly obtained by one-step reactions between aromatic aldehydes and primary aromatic amines. These one-step reactions may be carried out in refluxing ethanol [46], in refluxing toluene in a Dean–Stark apparatus [47] or in water at 25 °C during 2 h [48] or 3 days [33] (Scheme 1). The reagents used to carry out these straightforward condensation reactions are mostly commercially available allowing to obtain large series of aza-stilbenes, most of them being substituted with hydroxyl groups.

**Scheme 2:**
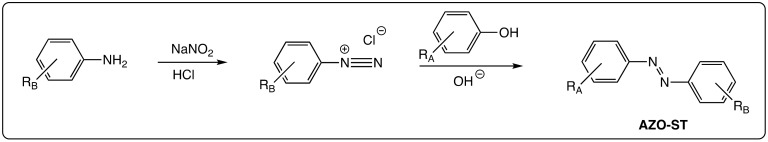
Synthetic methods for obtaining Azo-stilbenes (AZO-ST).

In contrast, the synthesis of azo-stilbenes (AZO-ST) requires several steps: preparation of a diazonium salt from an aromatic primary amine followed by diazo-coupling reaction between this salt and an aromatic compound [32,49] (Scheme 2). As in the case of AZA-ST, most AZO-ST are substituted with hydroxyl groups. Thus, the library of AZO-ST is large because of the commercial availability of the reagents.

However, reported works about aza-stilbenes are more numerous than those concerning azo-stilbenes, probably because of their asymmetrical double bond which may afford more structural, chemical and biological specifications.

### 2.3. Symmetry or Dissymmetry of Double Bonds

**Scheme 3:**
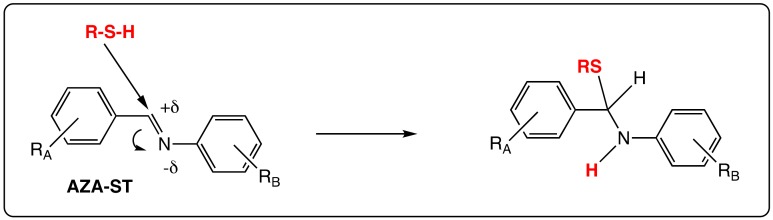
Example of the nucleophilic attack of a thiol on an imino bond in AZA-ST.

The N=N bond in AZO-ST is symmetric as in the case of RSV and does not provide an electronic influence on one or the other of the aromatic rings, especially on their substituents. In contrast, the presence of imino bond C=N in AZA-ST induces a dissymmetry inside the stilbene core. According to the number and positions of hydroxyl groups on both aromatic rings A and B (Figure 2), their position with respect to the nitrogen atom in the linkage, mono or poly hydroxy AZA-ST may give rise to various biological studies. Indeed, the lone electronic pair of the nitrogen atom can play a role in the stabilization of a phenoxyl radical or may allow an intramolecular hydrogen bond. In addition, the imino bond is polarized and the carbon atom may be attacked by a nucleophilic agent, such as a thiol group of a cysteine residue. These features that do not appear in the case of RSV impute to aza-stilbenes specific behaviors in biological environment (Scheme 3).

## 3. Biological Activities of Aza-Stilbenes

### 3.1. Aza-Stilbenes Bearing A Hydroxyl Group in Ortho Position of Cycles A or/and B

**Figure 4:**
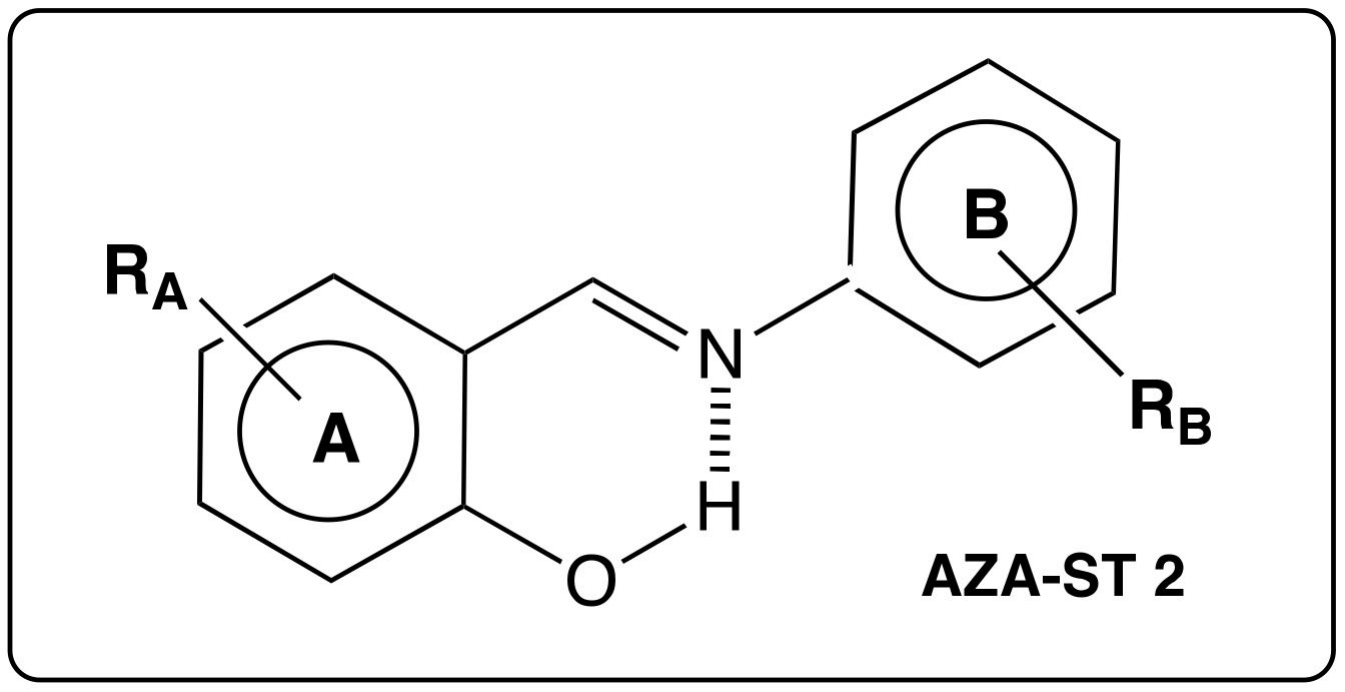
AZA-ST **2** bearing OH group in ortho position in ring A [48].

Anti-oxidant activity is widespread in polyphenols and especially in the case of resveratrol. At first, the comparison between RSV and its imino analogs has been focused on anti-oxidant and radical scavenging activities by different research groups. The high anti-oxidant activities of AZA-ST **2** towards 2,2-diphenyl-1-picrylhydrazyl (DPPH) radical and galvinoxyl (GO) radical have been explained by the acidity of the phenolic proton due to an intramolecular hydrogen bond between this phenolic proton and the nitrogen atom in the imine group (Figure 4) [48]. In addition, the ^1^H-NMR spectrum of **2** showed high chemical shifts for such phenolic protons (δ = 12.25 to 14.19 ppm) that are typical of acidic protons.

The authors have suggested the following mechanism in three steps [48]. The spontaneous release of the phenolic proton leading to the formation of a phenolate anion may trigger the anti-oxidant mechanism in AZA-ST **2** (ArOH → ArO^−^ + H^+^). Indeed, the phenolate anion loses an electron in favor of a free radical R^•^ (ArO^−^ + R^•^ → ArO^•^ + R^−^) especially since the phenoxyl radical is stabilized on the aromatic ring and anion R^−^ is neutralized by proton provided in the first step (R^−^ + H^+^ → R − H).

**Figure 5:**
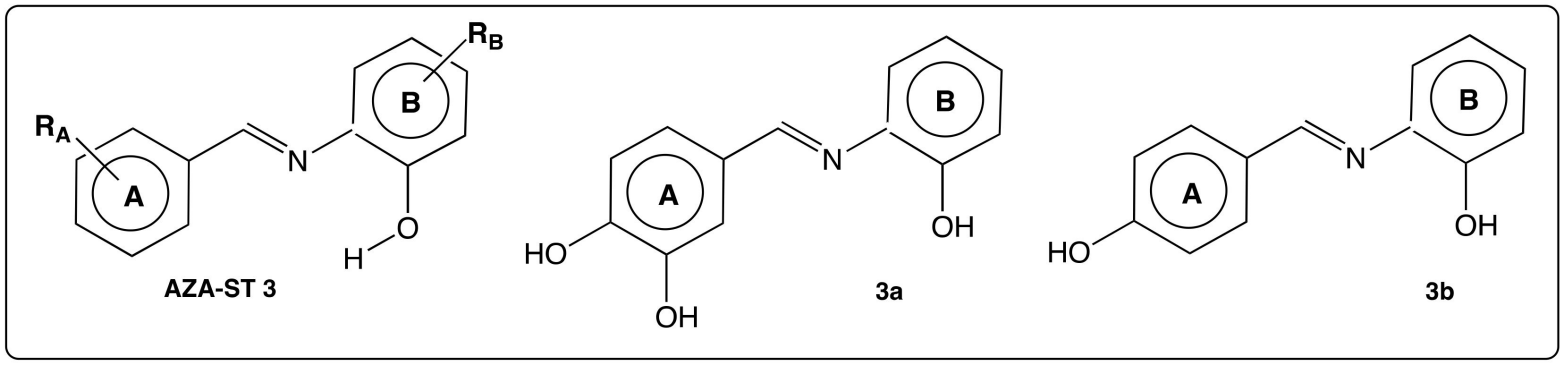
Structure **of** compounds of AZA-ST series **3** and AZA-ST **3a** and **3b** bearing OH in *ortho* position in ring **B** [51].

Radical scavenging activities (RSA) of compounds of AZA-ST series **3** (Figure 5) against DPPH radical have been studied [50,51]. Imino RSV analogs **3** bearing a hydroxyl group in ortho position of cycle B have shown a better activity than the parent molecule [51]. The lone electronic pair of nitrogen atom would overlap with the phenoxyl radical at *ortho* position of the cycle B, initiated by the scavenging of DPPH radical. On the other hand, the presence of a catechol group on cycle A in AZA-ST **3a** increased the anti-oxidant activity because the resulting phenoxyl radical may be stabilized by resonance and formed subsequently a *o*-quinone [52]. Indeed, compound **3a** was the most effective anti-oxidant agent of this series against DPPH radical and provided an IC_50_ value (expressed in µM) 6-fold lower than RSV. In addition, compounds in the AZA-ST series **3** were evaluated for their ability to quench singlet oxygen ^1^O_2_ by using EPR spin-trapping technique. All of them appeared to be better quenchers than RSV, especially compound **3b**, whose IC50 value (expressed in µM) was 15-fold lower compared to that of the parent molecule [51]: the IC50 of compound **3b** determined with the EPR spin-trapping technique was (0.99 ± 0.06) µM whereas the IC50 value of RSV was (16.94 ± 0.73) µM.

**Figure 6:**
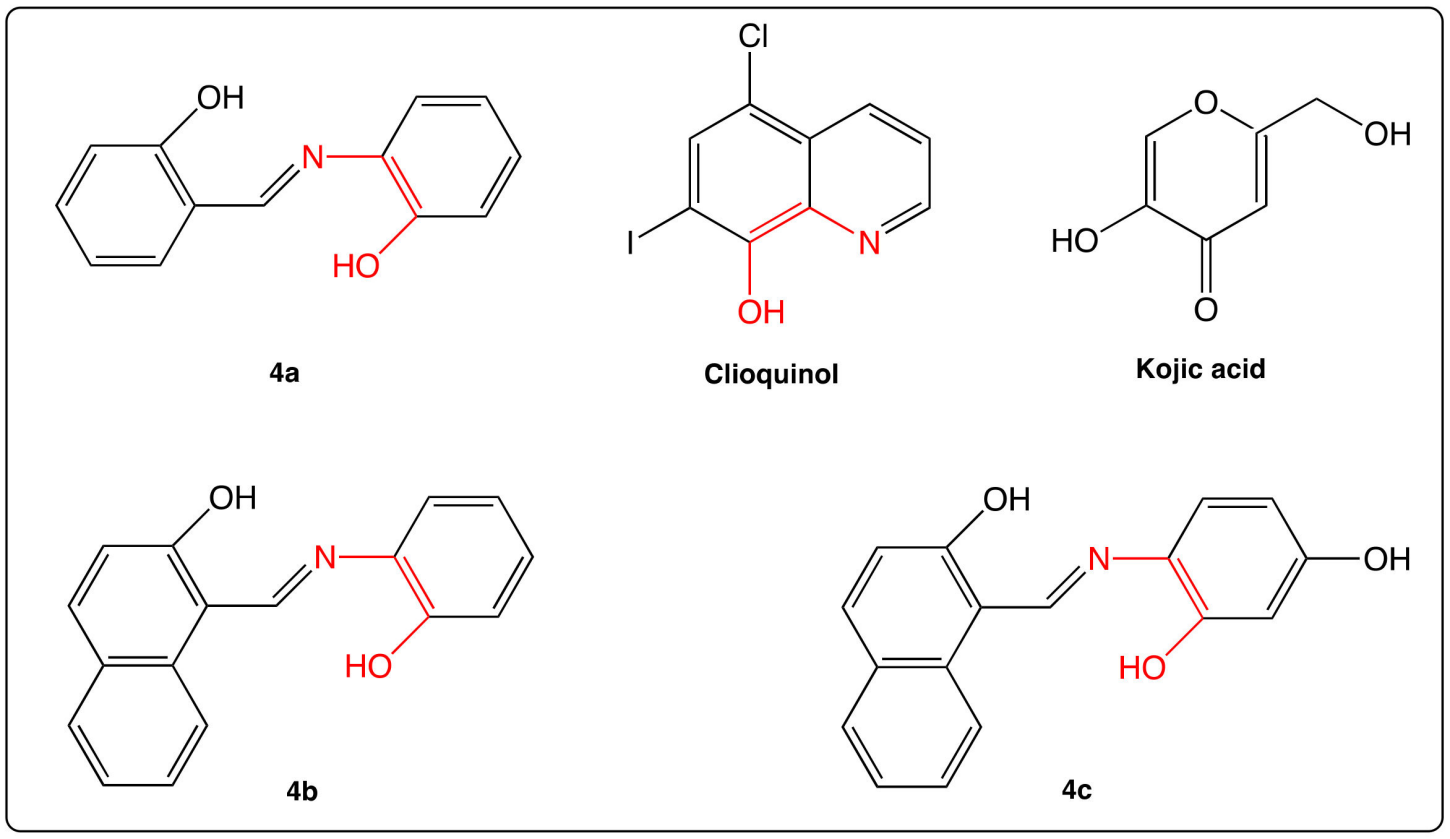
Structure of clioquinol, kojic acid and AZA-ST **4a**–**c**.

In addition to anti-oxidant features of imino RSV analogs, Zhang’s group has shown a correlation between radical scavenging activities of different aza-stilbenes and their abilities to chelate transition metal ions such as Cu^2+^ and Fe^3+^, especially in the case of AZA-ST **4a** (Figure 6) [53]. The authors have suggested that the “N=C=C-OH” sequence (shown in red in Figure 6) was a “metal ion-binding motif”.

Free radicals and transition metal ions including Cu^2+^, Fe^2+^ and Fe^3+^ catalyze oxidative damages as does the Fenton reaction (Fe^2+^ + H_2_O_2_ → Fe^3+^ + OH^−^ + HO^•^) in some age-related diseases by initiating decomposition reactions of hydrogen peroxide (H_2_O_2_) with metal ions to generate the hydroxyl radical (HO^•^), which is a powerful pro-oxidant. Therefore, the AZA-ST **4b**–**c** (Figure 6) were designed by conjugation of RSV and clioquinol, both compounds being different agents against Alzheimer’s disease. Indeed, in vitro, RSV is known for its inhibition of the aggregation of amyloid-β (Aβ) [54,55], the main component of amyloid plaques in Alzheimer’s diseases; clioquinol, bearing a metal ion-binding motif, is fit to slow down the neurological decline in early stage clinical trials [56]. To combine both features and strengthen the activity against Alzheimer’s disease, ionophoric polyphenols **4b** and **4c**, bearing a hydroxyl group in *ortho* position of both aromatic cycles, were synthesized [57]. The *ortho* position of hydroxyl group on ring B was essential to keep the same ability than clioquinol to chelate Cu^2+^ for an efficient activity. In contrast, the involvement of the lone electronic pair of nitrogen in the metal complexation prevents intramolecular hydrogen bonding with hydroxyl group in position *ortho* of cycle A as in the case of AZA-ST **2** [48]. However, this phenolic group is crucial for scavenging free radicals produced during the interaction between abnormal amyloid-β (Aβ) and Cu^2+^ [58].

AZA-ST **4a** has been evaluated for its ability to inhibit tyrosinase. Indeed, tyrosinase is a copper-containing protein; it is implied in the melanin biosynthesis in melanocytes and subsequently in hyperpigmentation of the skin. Chelators of copper ions, such as kojic acid (Figure 6) are good candidates to inhibit tyrosinase action [59]. Given its feature to bind Cu^2+^ ion [53], AZA-ST **4a** (Figure 6) was evaluated for its ability to inhibit tyrosinase by Lima’s group [60]. Compound **4a** turned out to provide a lower tyrosinase inhibitory activity than kojic acid; however, compound **4a** showed a better depigmenting activity than RSV.

**Figure 7:**
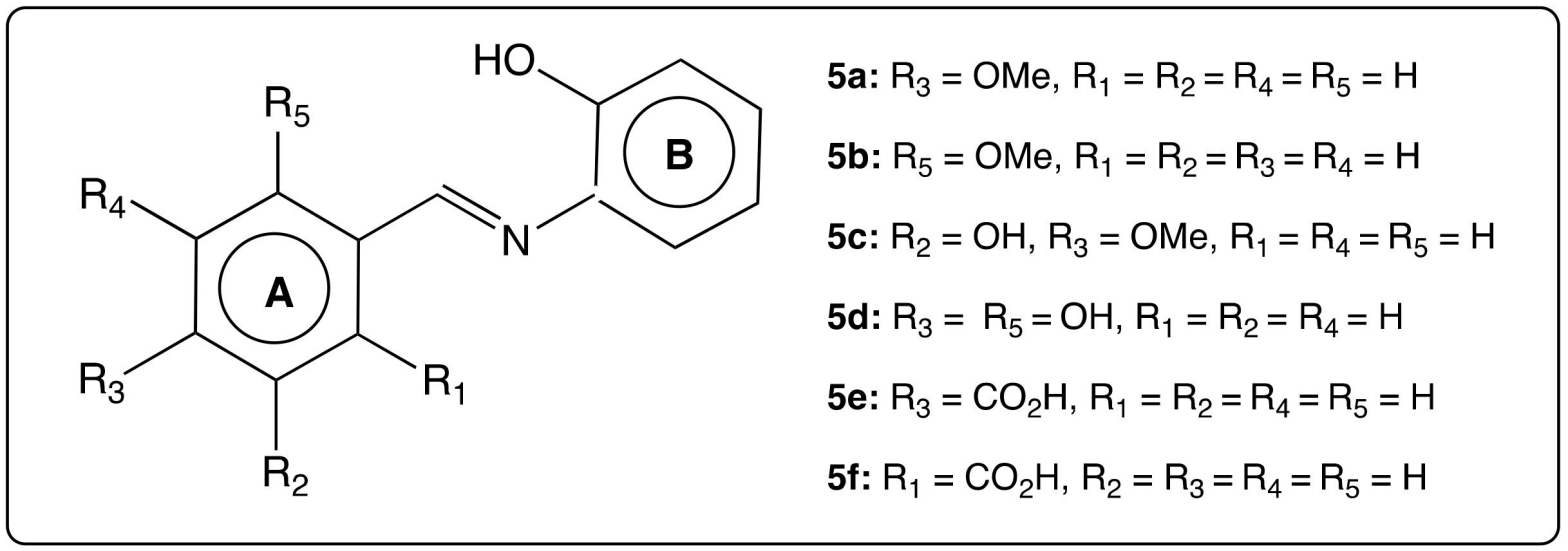
Structure of the compounds of the AZA-ST series **5** (AZA-ST **5**) [61].

The compounds in the compounds of the AZA-ST series **5** (Figure 7) were tested for their antioxidant activities and compared to RSV [61]. They were more effective DPPH radical scavengers than RSV. In addition, compounds **5a** and **5e** turned to provide anti-inflammatory properties.

**Figure 8:**
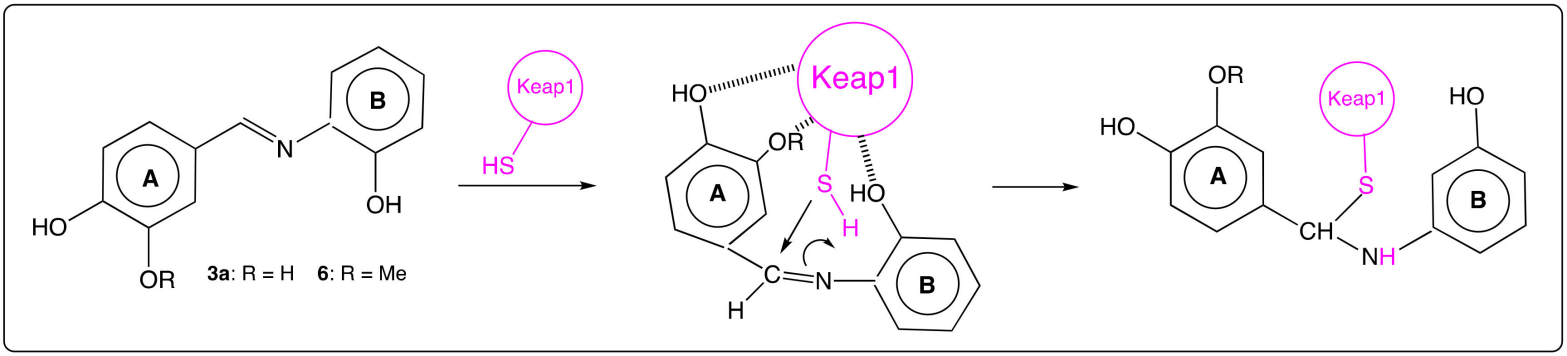
Suggested mechanism in the adduct of Keap1 protein on AZA-ST **3a** and **6**. (Inspired from [33]).

Apart from the fact that the lone pair of nitrogen atom may play crucial roles in anti-oxidant activities and metal chelation, polarization of the imino bond providing an electrophilic character to the carbon atom is essential in reactions with nucleophilic agents, such as a thiol function of the cysteine residues in proteins. Among such proteins, Keap-1 (Kelch-like ECH-associated protein 1) is a major repressor of Nrf2 (Nuclear factor erythroid-2-related factor 2), a transcription factor regulating the expression of several genes encoding for enzymes involved in the control of RedOx homeostasis. Indeed, Keap-1 may form a complex with Nrf2, that prevents the transcription factor to bind to antioxidant response elements (ARE) in the nucleus. Li’s group has shown the ability of AZA-ST **3a** and **6** (Figure 8) to activate Nrf2 and proposed the following mechanism: Keap-1 is kept close to AZA-ST **3a** or **6** by interactions between the protein and hydroxyl (or methoxyl for **6**) groups in *para* and *meta* positions of cycle A and *ortho* position of cycle B [33]. These interactions give a conformation to the aza-stilbene as the thiol group of cysteine residue may attack the carbon atom of the imino linkage to form a covalent bond and cause the release of Nrf2 (Figure 8) [33]. In this case, hydroxyl groups of both aromatic cycles are involved either upon interactions between the lone pairs of oxygen atoms and Keap-1 either by intramolecular hydrogen bonds with this protein.

**Figure 9:**
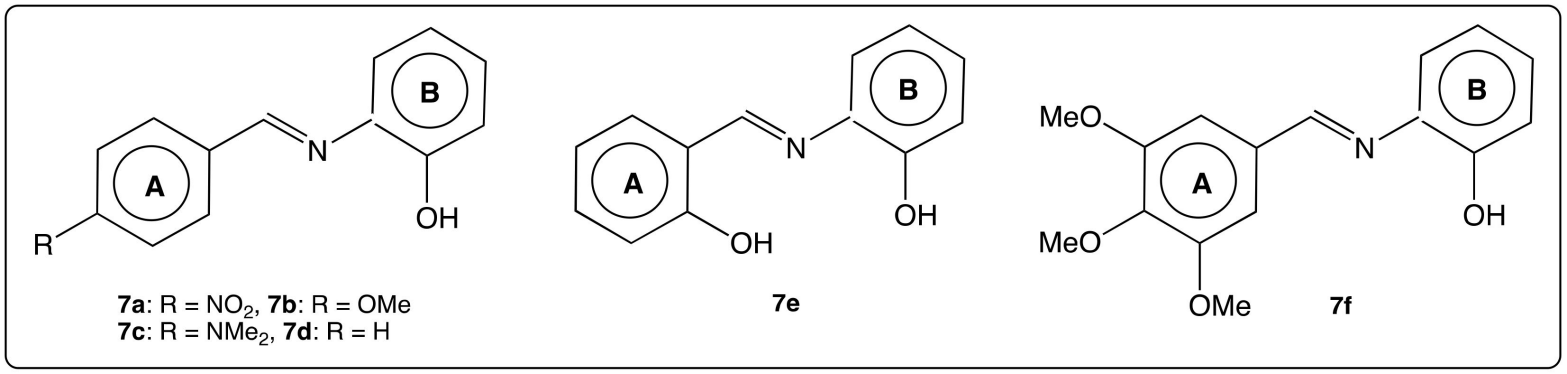
Structure of AZA-ST **7a**–**f** [62].

Other compounds of a new AZA-ST series **7** (Figure 9) bearing a hydroxyl group in the *ortho* position of cycle B were synthesized and have shown promising anti-leishmanicidal and antituberculosis activities [62]. The authors suggest that activity of compounds **7a**–**7f** (which appear to be moderate) depends on the electronic density of the substituent in *para* position of cycle A. In contrast, the role of the hydroxyl group in cycle B was not mentioned.

### 3.2. Aza-Stilbenes Bearing A Catechol Group on Cycle A

**Figure 10:**
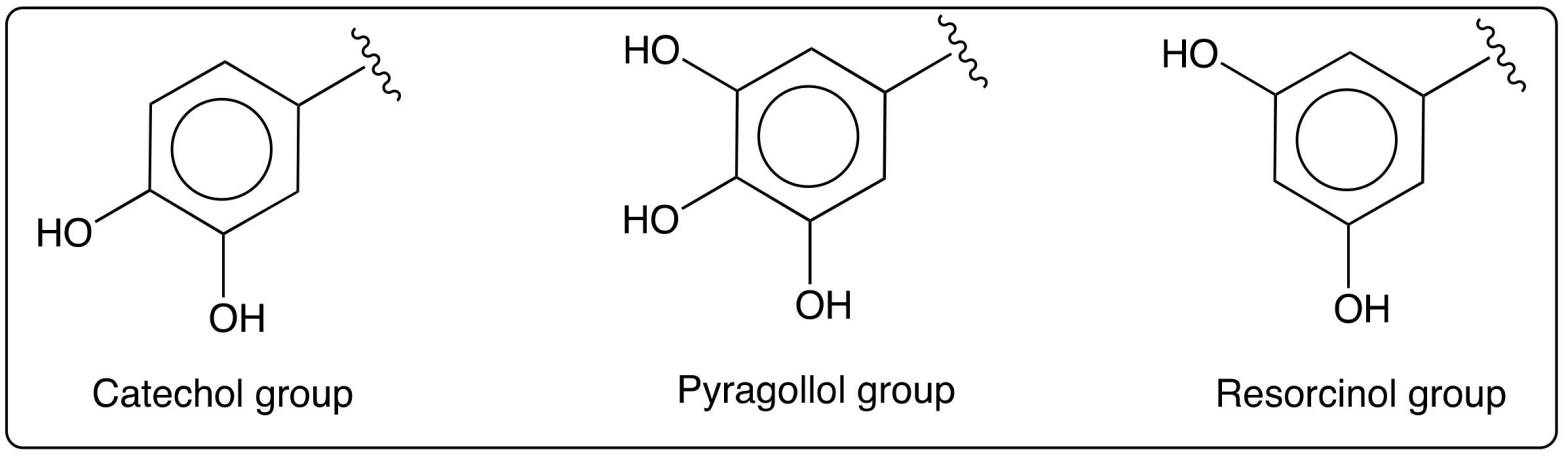
Catechol, pyrogallol and resorcinol groups [55].

Murias’s group has reported several studies on polyhydroxylated stilbenes bearing catechol or pyragollol groups on one or both aromatic cycles. They have highlighted the crucial roles of such groups in anti-oxidant activities and subsequently anti-tumoral activities of such polyhydroxylated stilbenes compared to that of RSV, which bears a resorcinol group (Figure 10) [63]. Indeed, as it was demonstrated by Wright [52], a phenoxyl radical formed from a catechol group may rise to a stabilized *ortho*-quinone, that it is not achievable with a resorcinol group. Thus, polyhydroxylated stilbenes bearing a catechol or a pyrogallol group provide better antioxidant and antitumoral activities than RSV.

**Figure 11:**
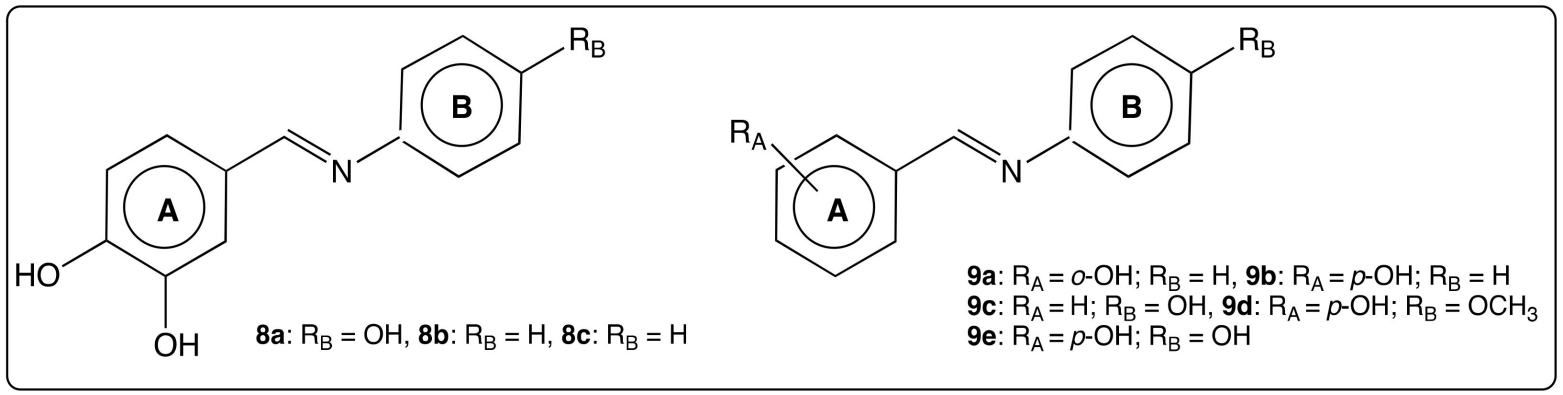
Structure of AZA-ST **8a**–**c** and **9a**–**e** [64].

In the case of catecholic aza-stilbenes, we have mentioned the above example of aza-stilbene **3a** (Figure 5) providing efficient anti-oxidant activities [51]. However, this feature was attributed to both the catechol group on cycle A and the phenol group in *ortho* position of cycle B. In the following examples, it will be shown that only catechol group on cycle A may be responsible for biological activities. Catechol compounds AZA-ST **8a**–**b** and phenol compounds AZA-ST **9a**–**e** (Figure 11) were synthesized and evaluated for their ability to scavenge GO radical [64]. The authors have shown that GO radical scavenging reaction rates of **8a**–**b** were higher than those of RSV and **9a**–**e**. Lu’s group also reported a weak DPPH radical scavenging activity for AZA-ST **9a**–**e** [51]. In addition, **8a**–**b** provided better antiproliferative activity against human hepatoma HepG2 cells than RSV and **9a**–**e** with IC_50_ values 14-fold and 11.7-fold lower than RSV IC_50_ values, respectively. Regarding catecholic AZA-ST **8a**–**b**, the results highlight a correlation between anti-oxidant and anti-proliferative activities as well as the crucial role of the catechol group in anti-oxidant activities of such RSV analogs.

Bhat’s group has reported the ability of AZA-ST **8a** and **8c** (Figure 11) to inhibit the growth of human breast cancer cell lines (MDA-MB-231, which is estrogen receptors (ER) negative and expresses mutated p53, and T47D, which is ERα positive) [46,65,66]. A docking study has allowed to show that (thanks to catechol groups) Van der Waals bonds might be promoted between the amino acid residues of protein in receptor ERα cavity and AZA-ST **8a** and **8c**. They would lead to a better stabilization of the imino RSV analogs into the protein pocket than RSV itself [46]. In subsequent studies, the same authors have highlighted that AZA-ST **8a** and **8c** might act on both estrogen receptors by inhibiting ERα expression and promoting ERβ expression [65,66].

## 4. Biological Activities of Azo-Stilbenes AZO-ST

**Figure 12:**
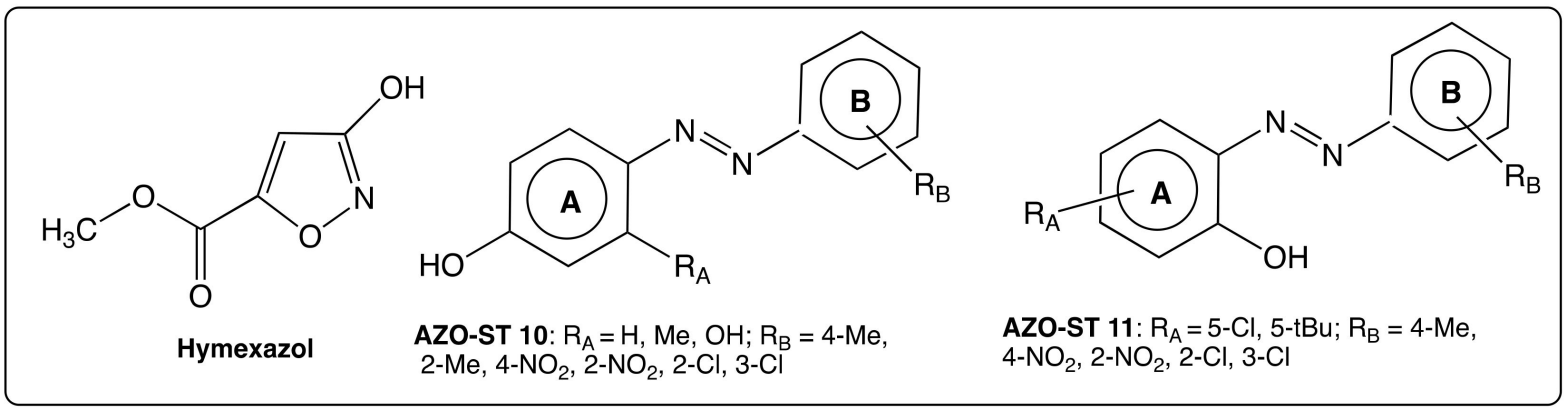
Structure of hymexazol and structure of compounds of the AZO-ST series **10** and AZO-ST series **11** [67].

Two series of azo-stilbenes AZO-ST **10** and AZO-ST **11** (Figure 12) were synthesized and evaluated in vitro for their antifungal activities against seven phytopathogenic fungi [67]. AZO-ST **11**, bearing a hydroxyl group in the *ortho* position of aromatic cycle A were less efficient than hymexazol the commercially agricultural fungicide (Figure 12). In contrast, AZO-ST **10** was efficient towards such fungi; some of them were more efficient than hymexazol. The substitution by a hydroxyl group in the *para* position of the aromatic cycle A and additionally by a methyl group in the *ortho* position of cycle A, seemed to be crucial structural parameters for AZO-ST **10** to get promising antifungal properties.

**Figure 13:**
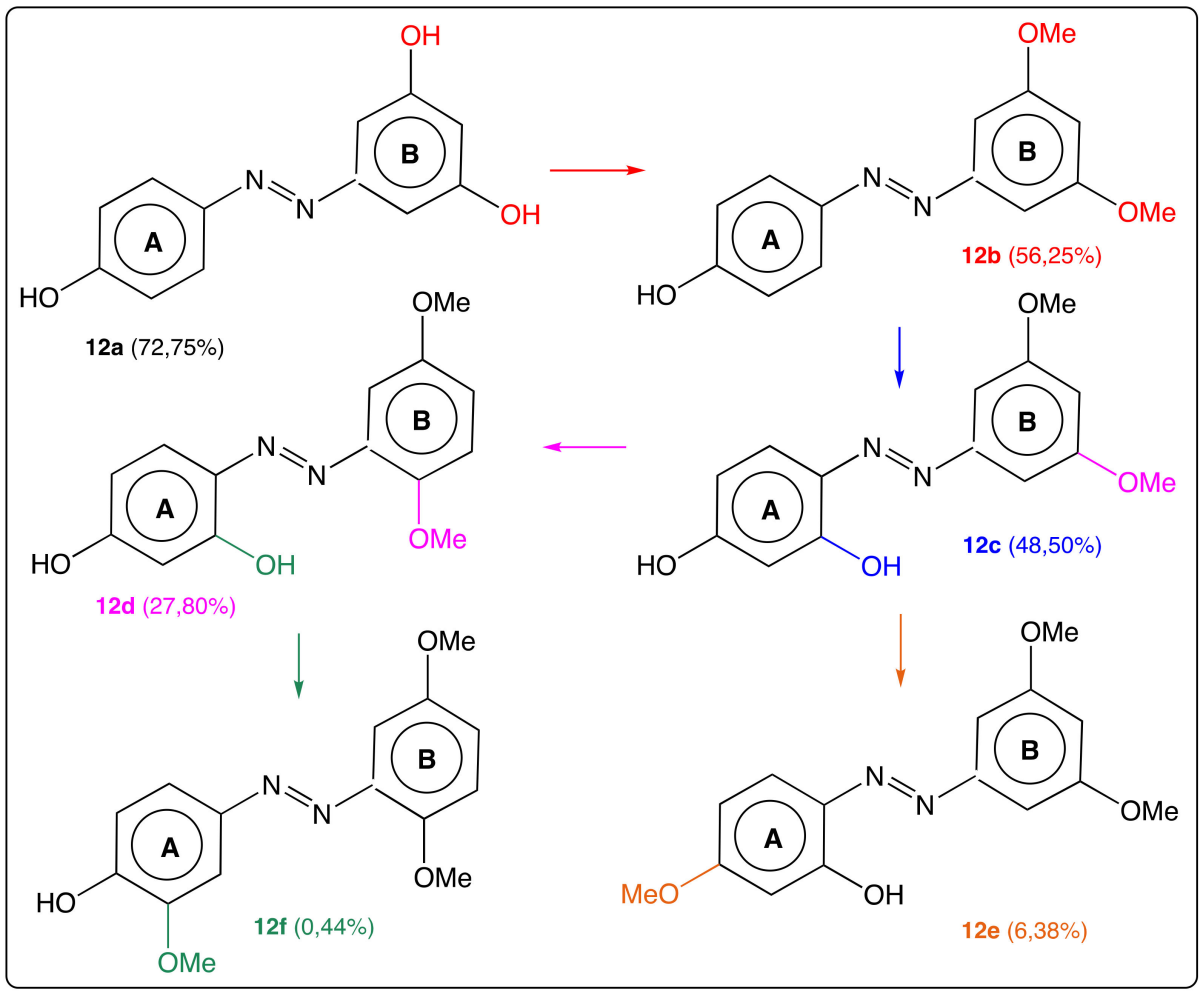
Structure of AZO-ST **12a**–**f** (and percentage of tyrosinase inhibition) [49].

Azo-stilbenes AZO-ST **12** (Figure 13) substituted with hydroxyl or methoxyl groups were synthesized and tested in vitro as potent tyrosinase inhibitors [49]. Azo-resveratrol (**12a**) and azo-pterostilbene (**12b**) with the *para* hydroxy group on cycle A had the best inhibition activities against mushroom tyrosinase in this series similar to those of RSV itself. In Figure 13, it is highlighted that the slightest modification of a group and of its position on both aromatic rings may have a great impact on the ability to inhibit tyrosinase. It can also be observed that even the induction of the *para* hydroxyl group is central, the role of the *ortho* hydroxyl group on cycle A is moderate and may be easily disturbed by a methoxyl group lying nearby.

**Figure 14:**
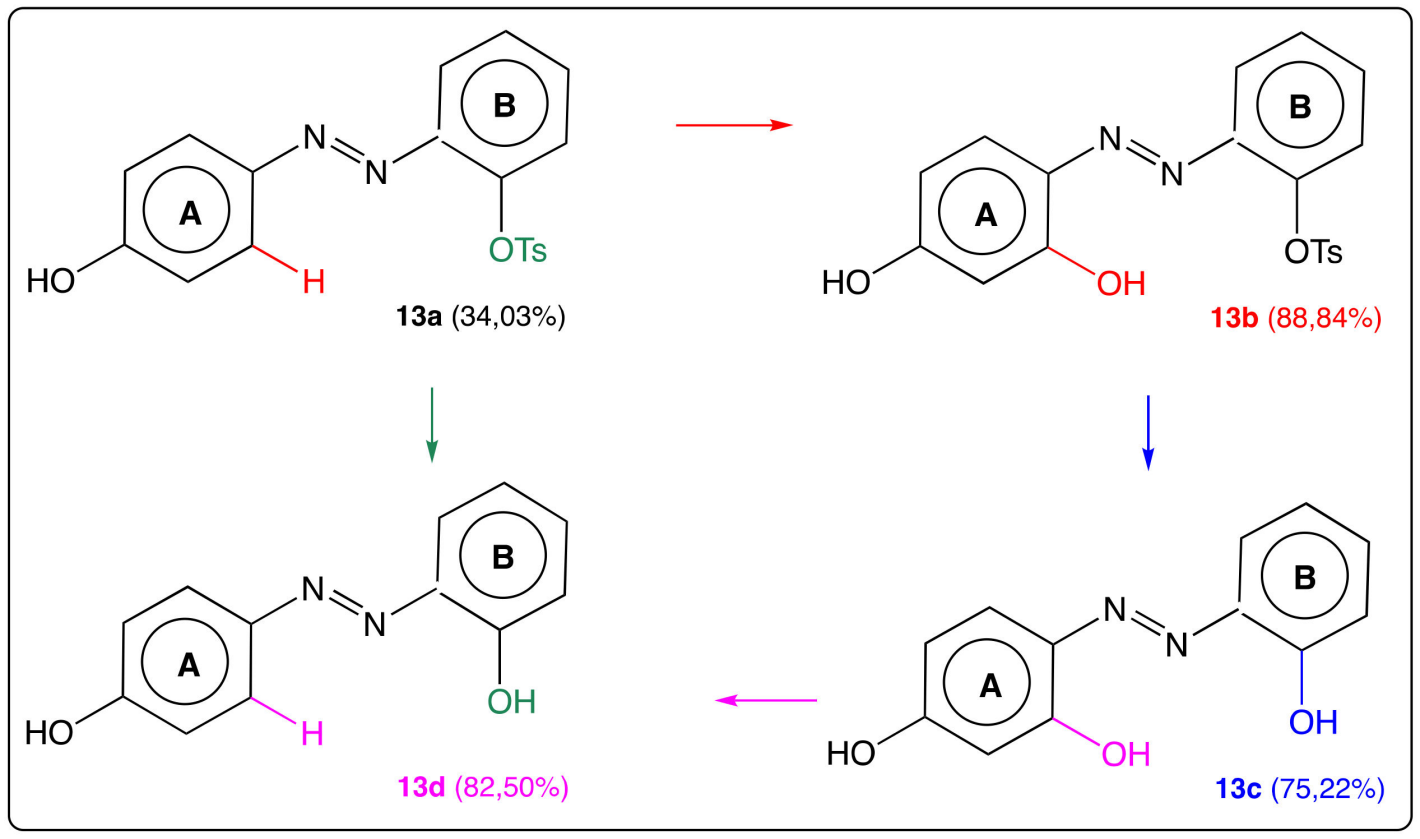
Structure of AZO-ST **13a**–**d** (and percentage of tyrosinase inhibition) [32].

The same research group has refined the structure of the compounds of the AZO-ST series **12** taking into account both important effects of the *para* hydroxyl group and the moderate effect of the *ortho* hydroxyl group in cycle A in the inhibitory activity on mushroom tyrosinase [32]. Four new compounds in the AZA-ST series **13a**–**d** (Figure 14) were synthesized, evaluated *in vitro* as potent mushroom tyrosinase inhibitors and compared with kojic acid (Figure 6) and RSV. Overall, compounds **13a**–**d** are more active than both reference compounds and IC_50_ values are 17.85, 49.08 and 59.80 μM for **13b**, kojic acid and RSV, respectively. Tyrosinase exists widely in bacteria, fungi, plants, insects, vertebrates and invertebrates and is the rate limiting enzyme in the biosynthesis of melanin pigments responsible for colors in hair, skin and eyes. In such series, there are no more methoxyl groups whereas the number of hydroxyl group drops while two compounds bear a tosyl-oxy group. Thus, molecular structures being more stripped towards those of AZA-ST **12**, it is easier to see the influence of *para* and/or *ortho* hydroxyl groups. The weak percentage of tyrosinase inhibition obtained with **13a** shows that the combination of *para* and *ortho* hydroxyl groups is required to get potent tyrosinase inhibitors and subsequently, promising therapeutic agents to treat skin diseases (hyperpigmentation, lentigo, vitiligo and skin cancers) [68].

## 5. Conclusions

AZA-ST and AZO-ST are isosteric RSV derivatives in which the lone electronic pair(s) of nitrogen atom(s) bring different electronic effects in the molecular structure compared to that of RSV. In the case of aza-stilbenes, the dissymmetric imino linkage induces a polarization of this bond and subsequent nucleophilic attacks [33]. The replacement of one or both carbon atoms of the double bond between the mono- or poly-hydroxylated aromatic rings provides attractive biological properties to AZA-ST and AZO-ST such as antioxidant activities [51,64]. Some of them may be promising candidates to treat diseases whose current treatments are either non-existent or weakly efficient especially breast cancer with ERα negative [46,65,66], leishmaniasis and tuberculosis [62] and skin diseases [68]. Finally, the compounds of AZO-ST series **10** and **11** also show greater anti-fungal activities than hymexazol, a commercially available agricultural fungicide [67]. Some diazo compounds are photoactivatable and may interact with proteins and nucleic acids. Hence, such features should be taken into account to avoid interferences with biochemical tests used to define their biological activities [69]. Given the easy syntheses and biological potential of AZA-ST and AZO-ST, the development of such isosteric RSV derivatives may be a perspective of interest to highlight promising therapeutic and fungicide agents.
